# Profiling of long non-coding RNAs in hippocampal–entorhinal system subfields: impact of *RN7SL1* on neuroimmune response modulation in Alzheimer’s disease

**DOI:** 10.1186/s12974-024-03083-x

**Published:** 2024-04-06

**Authors:** Hanyou Liu, Jingying Li, Xue Wang, Shiqi Luo, Dan Luo, Wei Ge, Chao Ma

**Affiliations:** 1 Department of Immunology, Institute of Basic Medical Sciences Chinese Academy of Medical Sciences, School of Basic Medicine Peking Union Medical College, Beijing, China; 2 Department of Human Anatomy, Histology and Embryology, Neuroscience Center, National Human Brain Bank for Development and Function, Institute of Basic Medical Sciences Chinese Academy of Medical Sciences, School of Basic Medicine Peking Union Medical College, Beijing, China

**Keywords:** Alzheimer’s disease, LncRNA, *RN7SL1*, Neuroimmune processes

## Abstract

Alzheimer’s disease (AD) is recognized as the predominant cause of dementia, and neuroimmune processes play a pivotal role in its pathological progression. The involvement of long non-coding RNAs (lncRNAs) in AD has attracted widespread attention. Herein, transcriptomic analysis of 262 unique samples extracted from five hippocampal–entorhinal system subfields of individuals with AD pathology and without AD pathology revealed distinctive lncRNA expression profiles. Through differential expression and coexpression analyses, we identified 16 pivotal lncRNAs. Notably, *RN7SL1* knockdown significantly modulated microglial responses upon oligomeric amyloid-β stimulation, resulting in a considerable decrease in proinflammatory cytokine production and subsequent neuronal damage. These findings highlight *RN7SL1* as an essential neuroimmune-related lncRNA that could serve as a prospective target for AD diagnosis and treatment.

**Supplementary Information:**

The online version contains supplementary material available at 10.1186/s12974-024-03083-x.

## Introduction

Alzheimer’s disease (AD), the predominant cause of dementia, poses an increasing threat to the ageing global population [1]. The pathology of AD primarily includes amyloid-β (Aβ) plaques and neurofibrillary tangles [2]. However, the specific mechanisms of AD remain unclear, and the current treatment options provide only symptomatic relief. Recent breakthroughs in clinical trials of anti-Aβ antibodies, such as aducanemab and lecanemab, underscore the critical need for novel interventions [3]. Further exploration of AD pathology in the human brain is imperative to advance the development of novel therapeutic interventions.

The human hippocampus, composed of distinct regions labelled cornu ammonis 1 (CA1) through CA4, constitutes a vital component of the hippocampal formation, along with the dentate gyrus and subiculum [4]. The hippocampus and entorhinal cortex (EC), which are involved in advanced brain functions, such as memory, learning, and cognition, typically exhibit histological alterations in the early stages of AD. The pathological changes associated with AD initially occur in the EC, and with disease progression, the hippocampus becomes increasingly susceptible to the impact of AD and atrophies [5]. Considering the important role of the hippocampal–entorhinal system in AD, researchers are increasingly focused on understanding how these regions communicate and regulate at the molecular level.

Long non-coding RNAs (lncRNAs), which are more than 200 nucleotides in length and lack protein-coding capacity, actively participate in gene expression regulation by engaging with transcription factors, chromosomal DNA, miRNAs, and mRNAs [6]. LncRNA expression reportedly displays tissue and cell-type specificity, with notably elevated expression levels in the brain tissue [7]. They are known to be pivotal contributors to normal neural development as well as to the onset and progression of neurodegenerative disease [8]. Dysregulation of lncRNA expression is associated with various neurodegenerative diseases, including AD. Certain lncRNAs, such as *BACE1-AS* [9, 10], *NEAT1* [11, 12], *17 A* [13], and *51 A* [14], have been reported to be upregulated in AD compared to controls, and are involved in the regulation of AD pathology. In the study of AD mechanisms, lncRNAs are implicated in processes such as the production and accumulation of Aβ, neuroinflammation, and dysfunction of neurons and synapses [15], highlighting their potential as important molecules for the investigation, diagnosis, and treatment of AD. Exploring the lncRNA profiles in the subfields of the hippocampal-entorhinal system among individuals with AD pathology could offer more nuanced insights into AD pathology and treatment.

Recent investigations have underscored the critical role of immune system dysregulation in the pathogenesis of AD. Both central and peripheral immune responses are implicated in AD pathology, preceding the formation of Aβ plaques and tau neurofibrillary tangles [16]. Moreover, neuroinflammation, which is commonly associated with the persistent activation of microglia and astrocytes, is involved in AD pathogenesis [17–19]. Among innate immune cells, microglia, the tissue-resident macrophages of the central nervous system (CNS) and key contributors to neuroinflammation, play crucial roles in CNS maintenance, pathogen defence, and injury response [20]. In AD, the presence of Aβ activates microglia, which is initially beneficial for Aβ clearance [21], but prolonged activation leads to reactive microgliosis, causing Aβ accumulation, sustained inflammation, and neuronal damage, which diminishes microglial efficiency, thereby impeding Aβ breakdown [22, 23]. Overactivated microglia assume reactive states characterized by substantial phenotypic changes and elevated expression of proinflammatory cytokines, fostering a chronic neuroinflammatory environment that exacerbates neuronal and synaptic loss [24]. Inflammation induces dendritic spine loss via mechanisms such as caspase activation by reactive oxygen species from inflammatory cells [25, 26]. The loss of dendritic spines directly correlates with synaptic dysfunction and causes cognitive and memory impairments in AD [27]. Furthermore, single-cell RNA sequencing is a powerful method for revealing the heterogeneity of functional states within microglial populations. Keren-Shaul et al. identified disease-associated microglia (DAM) in AD mouse models and human postmortem AD brains [28]. DAM have been found to downregulate the expression of immunosuppressive genes and upregulate the expression of phagocytosis- and lipid metabolism-related genes, which are associated with AD risk factors such as Aβ plaques [29]. DAM highlights the specificity and complexity of microglia, necessitating further investigation to better understand the phenotypes and mechanisms of microglia under AD disease states, and suggesting the potential for the development of AD therapies.

Given the important role of neuroinflammation and microglial activation in AD pathology, it is essential to understand the alterations in neuroimmune responses and the functions of immune-related genes within the context of AD. In the present study, we performed transcriptomic sequencing of 262 unique samples from five subfields of the hippocampal-entorhinal system in postmortem human brains to explore changes in the expression of lncRNAs. Comprehensive bioinformatics analysis revealed alterations in lncRNAs and their potential functional roles. A key neuroimmune-related lncRNA *RN7SL1*, was identified and demonstrated a robust ability to differentiate between AD patients and healthy controls. Subsequent experiments indicated that *RN7SL1* affects microglial functions, including the expression of proinflammatory factors, phagocytosis, and migration, and influences neuronal apoptosis and morphology through microglia. Our findings underscore the involvement of *RN7SL1* in neuroinflammation, a critical aspect of AD pathology, suggesting its potential role in the pathogenesis and treatment of AD.

## Methods

### Human postmortem brain tissue collection

Human brain tissue samples were obtained from the National Human Brain Bank for Development and Function, Chinese Academy of Medical Sciences, and Peking Union Medical College, Beijing, China. Brain tissue collection adhered to international standard human brain banking procedures and occurred between 2012 and 2022. Prior to donation, all individuals provided informed consent for the utilization of their brain tissue in medical research. This study included 31 individuals with AD pathology and 22 age-matched control individuals without AD pathology. The AD pathology of each sample was assessed using the “ABC” score, as delineated in the guidelines by the National Institute on Aging and the Alzheimer’s Association [30]. Sequencing procedures were performed on the CA1, CA2, CA3, CA4, and EC subfields from these donors, yielding 262 samples in total. Postmortem brain case samples with associated traits are listed in Table S1 (Supplementary Material 2). Hippocampal dissections were performed using previously established protocols [31].

### RNA extraction, library preparation, and sequencing

Total RNA was extracted using the RNeasy Mini Kit (QIAGEN, 74106), as per manufacturer instructions. RNA integrity number (RIN) was determined using Agilent Bioanalyzer 2100 (Agilent Technologies). Only samples with a RIN value greater than 2 were included for further analysis. Library construction was performed using VAHTS Total RNA-Seq Library PrepKit for Illumina (Vazyme, NR603-02), according to manufacturer instructions. Subsequently, paired-end sequencing was performed on the Illumina HiSeq 2500 platform. Following sequencing, Seqtk (https://github.com/lh3/seqtk) was employed for preprocessing the reads, involving adaptor sequence trimming and low-quality read removal. Filtered reads were then aligned to the human genome hg38 using Hisat2 v2.0.4 [32], with the reference genome sourced from Ensembl (v79, GRCH38). StringTie v1.3.0 [33] was then utilized to count each gene following the alignment.

### Transcriptome data pre-processing

Subsequent analyses excluded lncRNAs with missing values or zero expression across all samples. Quantile normalization was applied to mitigate the influence of confounding variables, including experimental batches, RNA extraction, and scanning devices, on the experimental outcomes.

Principal component analysis (PCA) was conducted using the prcomp() function in R based on the counts per million of lncRNAs. PCA plot visualization was accomplished using the ggord R package [34].

Surrogate variable analysis [35] was performed using the sva R package to estimate hidden covariates, including age, postmortem interval (PMI), RIN, and area. The residuals from this analysis effectively eliminated the influence of these known covariates and any latent factors from the gene expression data.

### Differential lncRNA expression analysis

The DESeq2 R package [36] was employed to assess the differential expression of lncRNAs, with original count values for each lncRNA serving as input data. The “design” parameter in DESeq2 included diagnosis (pathological AD vs. control) as the primary factor of interest, with age, PMI, and RIN serving as covariates to account for potential impacts on gene expression.

The criteria for identifying differentially expressed lncRNAs (DELncs) were as follows: |log_2_ (fold-change)| > 0.58 and adjusted *P* < 0.05. Volcano plots generated using the plot() function in R visually represented the number of DELncs within each of the five subfields. In addition, a circular plot was generated using the circlize R package [37] to visualize the chromosomal distribution of differentially expressed genes, providing an overview of their genomic locations. A Venn diagram was constructed using the VennDiagram R package [38] to identify common and unique DELncs across the five subfields. Furthermore, a heatmap illustrating expression patterns and relationships of DELncs across the subfields was generated using the ggplot2 R package [39].

### Target gene prediction

A cis-regulatory prediction strategy was employed to predict potential target genes regulated by the identified lncRNAs. Genes located on the same chromosome within a genomic distance of 50 kb were considered as cis-target genes associated with the respective lncRNA.

To identify linearly co-expressed mRNAs, those displaying Pearson correlation coefficient > 0.8 when compared with the expression levels of the corresponding lncRNA across 262 samples were considered. The mRNA expression data used in this analysis were sourced from the study by Luo et al. [31].

### Defining neuroimmune-related lncRNAs (NILncs)

Pearson correlation analysis was performed to determine correlations between the expression profile of all lncRNAs and neuroimmune-related genes obtained from the same sequencing experiment as our lncRNAs [31]. LncRNAs displaying an absolute correlation coefficient of > 0.8 with at least one neuroimmune-related gene were defined as NILncs. Heatmaps illustrating correlations between the expression of lncRNAs and neuroimmune-related genes, along with the differential expression pattern of NILncs across the five subfields, were generated using the pheatmap R package. Besides, an UpSet plot was constructed using the UpSet R package [40].

### Target gene functional annotation and enrichment analysis

The classification of target genes included molecular function, cellular component, and biological process categories through gene ontology (GO) annotation. Enrichment analysis of GO terms was performed using the clusterProfiler R package [41]. Further, the potential involvement of lncRNA target genes in specific signal transduction or metabolic pathways was evaluated using the Kyoto Encyclopedia of Genes and Genomes (KEGG) database [42]. Visualization of the results obtained from GO and KEGG pathway enrichment analyses were performed using the ggplot2 R package.

### Weighted gene co-expression network analysis (WGCNA)

WGCNA was performed with the WGCNA R package using the processed lncRNA-seq dataset from 262 samples encompassing CA1–CA4 and EC regions sourced from 53 individuals [43]. A signed network using WGCNA was established, with the soft power parameter estimated and determined to be 14. A pairwise distance matrix based on the topological overlap measure was derived for selected genes. Co-expression modules (clusters) within the network were identified using a dynamic hybrid cut method, employing specific settings: minModuleSize, 30; verbose, 3; and resignment threshold, *P* < 0.05. Modules were randomly labeled with colors; lncRNAs not assigned to any module appeared in grey. The modulePreservation() function was used to assess the robustness of modules, determining Zsummary preservation scores via 200 permutations against each cohort tested using the consensus network as the template. A Zsummary score of > 10 indicated a strongly preserved module, < 2 indicated a non-preserved module, while a score between 2 and 10 indicated a moderately preserved module.

Node centrality, representing the sum of within-cluster connectivity measures, facilitated hub gene identification within each module. For visualizing the constructed networks, we applied hard thresholding to the edge distances and represented the networks using Gephi 0.9.2 [44].

### Cell-type and lncRNA set enrichment analysis

Cell-type-specific markers sourced from PanglaoDB [45] were utilized along with linearly co-expressed mRNAs to conduct Fisher’s exact tests. The aim was to explore the enrichment of specific cell types within lncRNA modules. Fisher’s exact test was also performed to assess the enrichment of the identified lncRNA sets, including DELncs and NILncs. Outcomes from the enrichment analysis were visualized using the circlize R package [37].

### Cell culture

Mouse BV2 microglial cell lines were cultured in Dulbecco’s modified eagle medium (DMEM; Hyclone, SH30243.01) supplemented with 10% fetal bovine serum (Gibco, A5669701). BV2 cells underwent regular passages every 2 days.

Primary microglia and neurons were isolated from postnatal day 1 Sprague-Dawley rats. Briefly, rat cerebral cortices were dissected in D-Hank’s balanced salt solution (Solarbio, H1D45) and enzymatically digested in 0.25% trypsin at 37 °C for 15 min. Subsequently, they were dissociated into individual cells through gentle pipetting and filtration using a 70-µm cell strainer. This cell suspension was centrifuged at 200 *g* for 5 min. For primary microglia culture, the cell suspension was seeded into a T75 flask and cultured in DMEM supplemented with 10% fetal bovine serum and 100 U/mL penicillin–streptomycin (Gibco, 15140122). After 3 days, the medium was replaced with one containing 20 ng/mL rat macrophage colony-stimulating factor (MCE, HY-P7386). After 3–4 days, microglia were isolated by shaking at 200 rpm for 2 h. For primary neuron culture, cells were resuspended in neurobasal medium (Gibco, 21103049) supplemented with 2% B-27 (Gibco, 12587-010), 0.5 mM L-glutamine (Gibco, 25030149), and 50 U/mL penicillin–streptomycin. These cells were then plated onto 24-well glass coverslips coated with poly-L-lysine (Sigma, P4707). The medium was changed every 5–7 days with 100 µL per well. All cells were maintained at 37 °C and 5% CO_2_.

### Oligomeric Aβ (oAβ) preparation

Aβ_1−42_ peptides (GL Biochem) were dissolved in 1,1,1,3,3,3-hexafluoro-2-propanol (Sigma, 1105228) at a final concentration of 1 mM. Upon natural evaporation of 1,1,1,3,3,3-hexafluoro-2-propanol in a fume hood, the peptides were dissolved in dimethyl sulfoxide (Sigma, D2650) to achieve a concentration of 1 mM. Aβ was then diluted to 10 µM with DMEM, followed by incubation at 4 °C for 24 h to obtain oAβ.

### Cell transfection and treatment

The lncRNA smart silencer (ssi) ssi- *Rn7sl1* and ssi-NC (negative control) were constructed by RiboBio Tech. Transfection of BV2 cells, primary microglia and primary neurons was performed using Lipofectamine 2000 (Invitrogen, 11668-019), as per manufacturer instructions. Following transfection, culture media was changed after 6 h. For quantitative PCR and conditioned medium stimulation experiments, after 24-hours of transfection, BV2 cells and primary microglia were treated with 2 µM oAβ for 12 h. For the phagocytosis assay, after 24 h of transfection, BV2 cells were stimulated with 5 µM oAβ for 24 h.

With regard to primary neurons (14 days in vitro), they were stimulated with 10 µM oAβ for 10 h or treated with 10 nM okadaic acid (OA; J&K Scientific, 288967) dissolved in dimethyl sulfoxide for 4 h; For the experiments involving oAβ stimulation and *Rn7sl1* knockdown, primary neurons were subjected to transfection for 24 h, followed by treatment with 10 µM oAβ for 6 h.

### Quantitative real‒time reverse transcription PCR (qRT‒PCR)

Total RNA from human brain EC tissues and cells was extracted using TRIzol (Invitrogen, 15596018) according to manufacturer instructions. Reverse transcription was performed using the PrimeScript RT Master Mix Kit (Takara, RR036A). RNA expression was determined by qRT‒PCR using TB Green Premix Ex Taq II (Takara, RR820A) on a Bio-Rad CFX96 system. For human brain tissues and cells, *GAPDH* and *β-actin* served as reference genes, respectively; relative gene expression was quantified using the 2^−ΔΔCt^ method. Primer sequences are listed in Table S2 (Supplementary Material 2).

### Phagocytosis assay

BV2 cells were treated with 0.01% (*v*/*v*) FITC-conjugated latex beads (Sigma, L1030) and 0.05% fetal bovine serum in DMEM at 37 °C for 4 h. For flow cytometry, BV2 cell pellets were collected by centrifugation at 300 *g* for 5 min and then resuspended in BD Cytofix™ Fixation Buffer (BD Biosciences, 554655). Subsequently, the cells were incubated for 10 min at 37 °C and ultimately resuspended in PBS. Flow cytometry was performed on a CytoFLEX flow cytometer (Beckman), collecting 30,000 cells per sample, and data was analyzed using FlowJo. Specifically, each sample file was loaded, and single-cell populations were gated based on forward scatter and side scatter properties to exclude debris and doublets. FITC^+^ cell populations were identified based on univariate histograms. The percentage of microglia that phagocytosed beads (% Phagocytosis) was calculated by dividing the number of FITC^+^ cells by the total number of gated cells.

### Transwell assay

BV2 cells treated with ssi- *Rn7sl1* and ssi-NC for 48 h were seeded within an 8-µm pore Transwell insert (Corning, 3422) in serum-free DMEM. DMEM containing 10 µM oAβ was then added to the receiver (i.e., bottom) chambers. BV2 cells were cultured for 24 h at 37 °C and 5% CO_2_. Subsequently, the upper surface cells were gently wiped away with a cotton swab, while the lower surface cells were fixed in 100% methanol for 30 min and then stained with 0.01% crystal violet for 2 h. Unbound crystal violet was removed by washing with ddH_2_O. To quantify the number of stained cells, the crystal violet on the cells was eluted with a 33% acetic acid solution at 100 rpm for 10 min, and absorbance of this solution was measured at 570 nm using a spectrophotometer.

### Primary neurons stimulated with microglial conditioned medium

Following transfection and oAβ stimulation, the supernatant from corresponding experimental groups of primary microglia was collected to generate microglial conditioned medium (CM). Primary neurons (21 days in vitro) were utilized, and neuronal culture media was replaced with CM for a 24-hours period.

### Immunofluorescence assay

Cells cultured on coverslips were fixed in 4% paraformaldehyde for 30 min and subsequently incubated with 0.3% Triton X-100 for 10 min. After blocking with 5% bovine serum albumin for 30 min, they were incubated overnight at 4 °C with primary antibodies: anti-IBA1 (1:500, Wako,019-19741), anti-NeuN (1:200, Abcam, ab104224), and anti-MAP2 (1:200, Cell Signaling Technology, 4542). Following primary antibody incubation, the cells were treated with Alexa Fluor 594 goat anti-rabbit IgG (1:200, Invitrogen, A-11,037) or Alexa Fluor 488 goat anti-mouse IgG (1:200, Invitrogen, A-11,029) for 1 h at room temperature. For dendritic spine staining, the cells were further stained with Alexa Fluor 488 phalloidin (1:500, Invitrogen, A-12,379) for 4 h at room temperature. Subsequently, nuclei were stained with 4′,6-diamidino-2-phenylindole (Solarbio, C0065) for 5 min. The cells were sealed using a fluorescent mounting medium (ZSGB-BIO, ZLI-9556) and then observed under a Leica DMi8 microscope or Leica Stellaris 5 confocal microscope. Three-dimensional reconstruction of dendritic spines and neuronal morphology was achieved using Imaris. Subsequently, statistical values were exported from Imaris. Among these, “Filament No. Sholl Intersections” were used for Sholl analysis. Sholl analysis graphs were created using the radius of concentric circles ranging from 0 to 300 μm and the number of intersections, with a step size of 20 μm. For TUNEL assay, cells were treated according to manufacturer instructions (RiboBio, C11012-1).

### Statistical analysis

Statistical analyses and data presentation were performed using R v4.1.0 and GraphPad Prism 9.0. A supervised machine learning technique, support vector machine (SVM) with a radial basis function kernel, was employed to develop a classifier based on lncRNAs, employing the e1071 R package [46]. The receiver operating characteristic (ROC) curve illustrates the trade-off between true (sensitivity) and false (1-specificity) positive rates at various classification thresholds. Area under the curve (AUC) values were computed by ROC curve analysis using the roc() function of the pROC package in R [47]. Values represent mean ± standard error of mean. Unpaired two-tailed Student’s *t*-test was applied to compare two experimental groups, while one-way analysis of variance (ANOVA) was used for multiple groups. *P* < 0.05 indicated statistical significance.

## Results

### Differential expression of lncRNAs in hippocampal–entorhinal subfields

After consolidating the bulk RNA-seq data from the five hippocampal–entorhinal system subfields, PCA was performed on the lncRNA sequencing results. PC1 and PC2 contributed significantly to the variance in the data (Supplementary Material 1: Fig. S1a). The PCA plot indicated a separation between pathological Alzheimer’s disease (AD) and control individuals (Supplementary Material 1: Fig. S1b). Subsequently, correlation coefficients between PCs and covariates were examined, revealing variable impacts of factors, including AD scores (Supplementary Material 1: Fig. S1c, d). AD scores exhibited the most significant impact, followed by subfield, age, PMI, and sex (Supplementary Material 1: Fig. S1d). To mitigate covariate effects, age, PMI, and RIN values were included for each of the five subfields in the differential expression analysis using DESeq2.

**Fig. 1:**
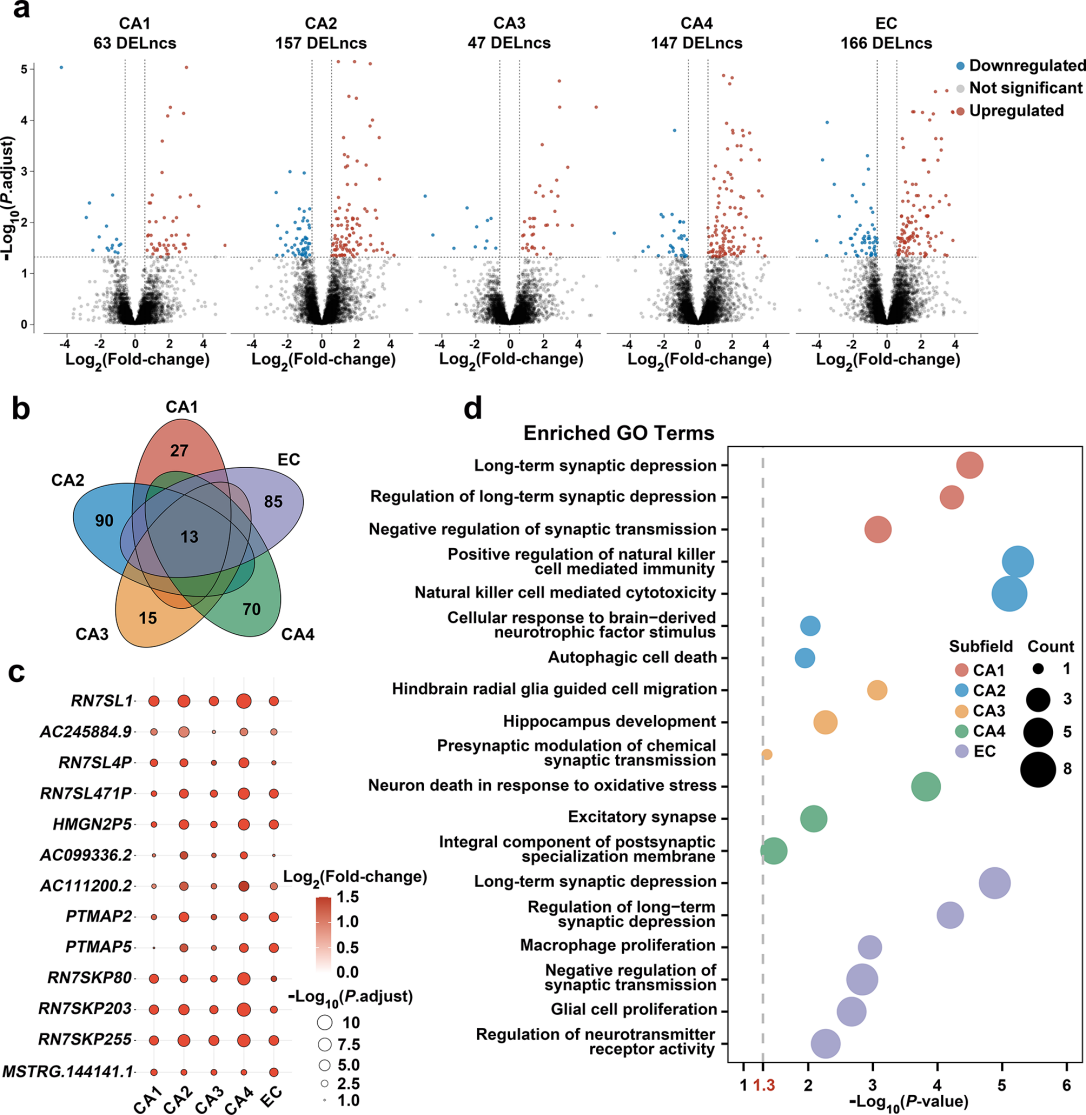
lncRNA expression pattern in the hippocampal–entorhinal system subfields of pathological Alzheimer’s disease (AD) and controls (without AD pathology). (**a**) Volcano plots of differentially expressed lncRNAs (DELncs) for each subfield. The criteria for identifying DELncs were |log_2_(fold-change)| > 0.58 and adjusted *P* < 0.05. Blue dots indicate downregulated lncRNAs, grey dots indicate lncRNAs with no significant changes, and red dots indicate upregulated lncRNAs. CA1–4, cornu ammonis subfields 1–4; EC, entorhinal cortex. (**b**) Venn diagram displaying the overlap of DELncs across five subfields, with 13 lncRNAs common to all subfields. (**c**) The extent of upregulation for the 13 common lncRNAs across all five subfields; the color represents the fold-change in pathological AD relative to controls, and the size of the circle represents the adjusted *P*-value (*P*.adjust). (**d**) Gene ontology (GO) enrichment analysis of cis-target genes of DELncs in each subfield

The differential expression analysis of the transcriptomes of individuals with AD pathology and those without AD pathology revealed 388 DELncs across all five subfields. Specifically, 63 DELncs were identified in CA1, 157 in CA2, 47 in CA3, 147 in CA4, and 166 in EC (Fig. 1a, Supplementary Material 2: Table S3). The distribution of DELncs across the chromosomes demonstrated a relatively uniform pattern (Supplementary Material 1: Fig. S2a). Among these DELncs, 13 were common across all five subfields, and their expression was upregulated in all subfields (Fig. 1b, c). Furthermore, substantial overlap and high correlation were observed among DELncs identified in pairwise comparisons between the five subfields (Supplementary Material 1: Fig. S2b).

To gain insights into potential biological functions, GO enrichment analyses were performed on the cis-target genes of DELncs specific to each subfield (Fig. 1d, Supplementary Material 2: Tables S4 and S5). A significant enrichment of terms related to synaptic function, cellular autophagy, neuroimmune processes, and glial cell function was observed. Notably, all of these terms are implicated in AD pathology [16, 48–51].

### Identification of NILncs through Linear correlation analysis between all lncRNAs and neuroimmune-associated genes

**Fig. 2:**
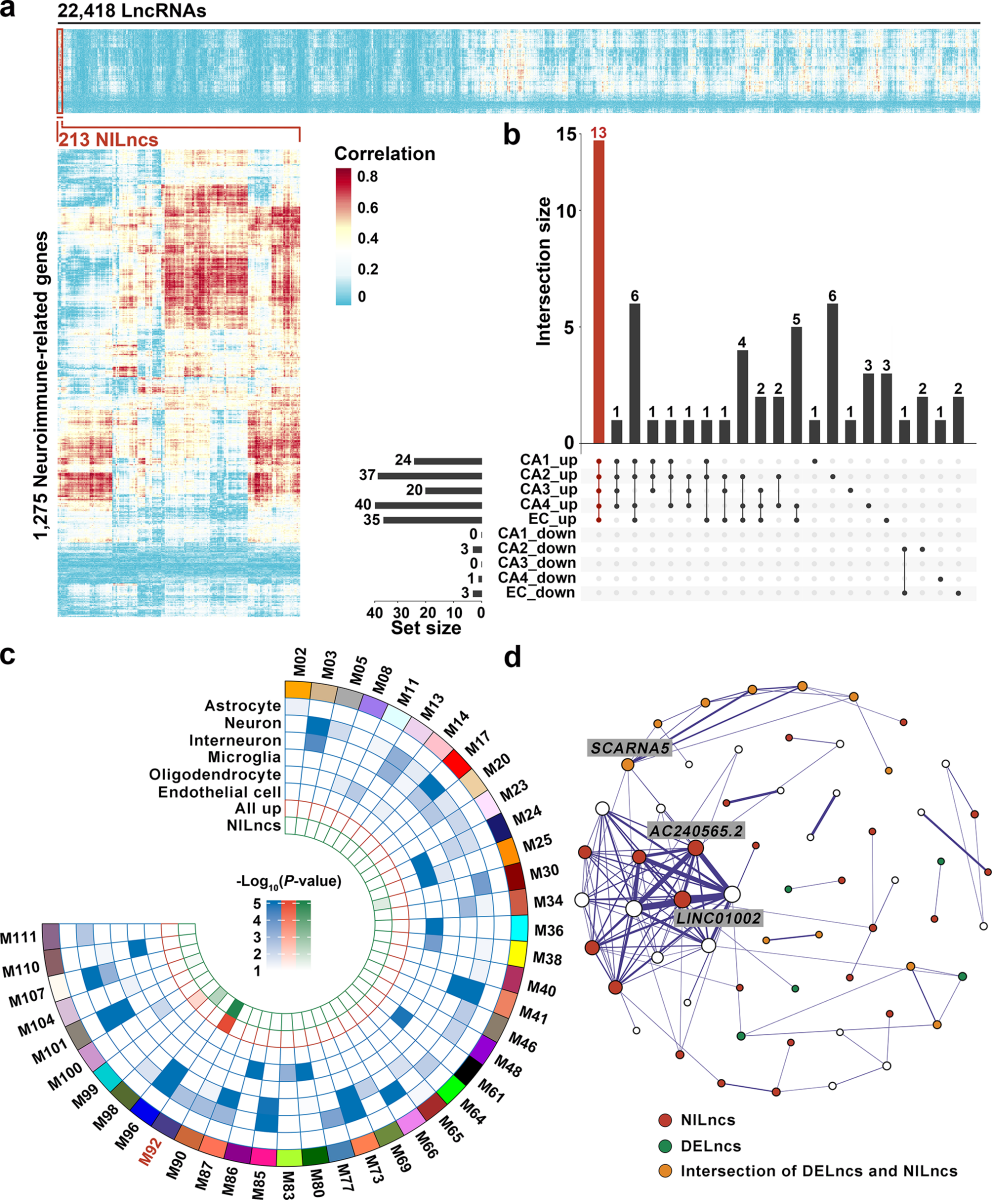
Identification of neuroimmune-related lncRNAs (NILncs) and weighted gene co-expression network analysis (WGCNA). (**a**) Heatmap depicting the linear correlation between neuroimmune-related genes and all lncRNAs, identifying 213 NILncs based on an absolute correlation coefficient of > 0.8 with at least one neuroimmune-related gene. (**b**) UpSet plot showing the overlap of differentially expressed NILncs among the five subfields. Linked dots indicate overlapping among 10 rows, which represent up-regulated or down-regulated lncRNAs in each subfield. (**c**) Degree of enrichment of cell-type-specific markers, DELncs, and NILncs across WGCNA modules, with colors representing corrected *P*-values from Fisher’s exact test. (**d**) Top WGCNA connections of WGCNA module M92. Node size represents the number of gene co-expression connections. Red: DELncs in any subfield; blue: NILncs; yellow: differentially expressed NILncs in any subfield

Considering the enrichment of neuroimmune-related terms among DELncs, we aimed to discern lncRNAs specifically associated with neuroimmune processes. For this purpose, a curated set of 1,275 neuroimmune-related genes was obtained from the study by Chen et al. [52]. Leveraging mRNA data from the same sequencing experiment as our lncRNAs [31], 213 NILncs that demonstrated a linear correlation with these neuroimmune-related genes were identified (Fig. 2a, Supplementary Material 2: Table S6). Notably, the majority of these NILncs exhibited upregulated expression patterns (Supplementary Material 1: Fig. S3a). Fifty-eight NILncs were also DELncs, including all 13 DELncs shared across the five subfields. The 58 NILncs that were differentially expressed among the five subfields are shown in Fig. 2b.

The identification of these 213 NILncs highlights their potential involvement in neuroimmune processes, signifying their possible relevance in the context of AD pathology.

### WGCNA

WGCNA was performed to uncover valuable insights into the expression profiles of 22,418 lncRNAs. Considering the absence of marked differences in expression patterns across the five subfields observed in PCA (Supplementary Material 1: Fig. S1e) and the high correlation among DELncs in these subfields (Supplementary Material 1: Fig. S2b), we aggregated data from all individuals across these subfields, resulting in the generation of 118 modules (Supplementary Material 1: Fig. S3b, Supplementary Material 2: Table S7). Ninety-three modules were shortlisted after a rigorous evaluation of module robustness using a stringent criterion (Zsummary score > 10) and were included in the following analysis (Supplementary Material 1: Fig. S3c).

To investigate the relationships between these modules and AD pathology, as well as their associations with specific cell types within the neural system, enrichment analysis was performed by intersecting linearly coexpressed mRNAs of the obtained modules with gene sets representing different cell types. Moreover, enrichment analyses were performed by intersecting the modules with DELncs and NILncs. The 43 modules that were significantly enriched (*P* < 0.05) with any of the lncRNA sets are shown in Fig. 2c.

One particular module, M92, exhibited notable enrichment of both DELncs and NILncs and was primarily enriched with neuronal marker genes, indicating a close potential association of M92 with neuroimmune processes and AD pathology. Further investigations employing GO and KEGG pathway enrichment analyses on the linearly coexpressed mRNAs with M92 lncRNAs revealed an association with neurodegenerative diseases, such as AD, synaptic function, glial cell activity, and cellular autophagy (Supplementary Material 1: Fig. S3d, Supplementary Material 2: Table S8).

An interaction network was constructed to visualize the core lncRNAs within M92 (Fig. 2d). Herein lncRNAs exhibiting a high level of connectivity within a module are referred to as hub lncRNAs are expected to possess functional significance within the module. Many hub lncRNAs were previously defined as NILncs, reinforcing the potential correlation between M92 and AD pathology as well as neuroimmune dysregulation. Notably, one of the hub lncRNAs, *LINC01002*, was implicated in a coding–noncoding gene coexpression network associated with periventricular white matter damage, suggesting its role in brain development [53].

### Identification of key lncRNAs and validation in an independent dataset

**Fig. 3:**
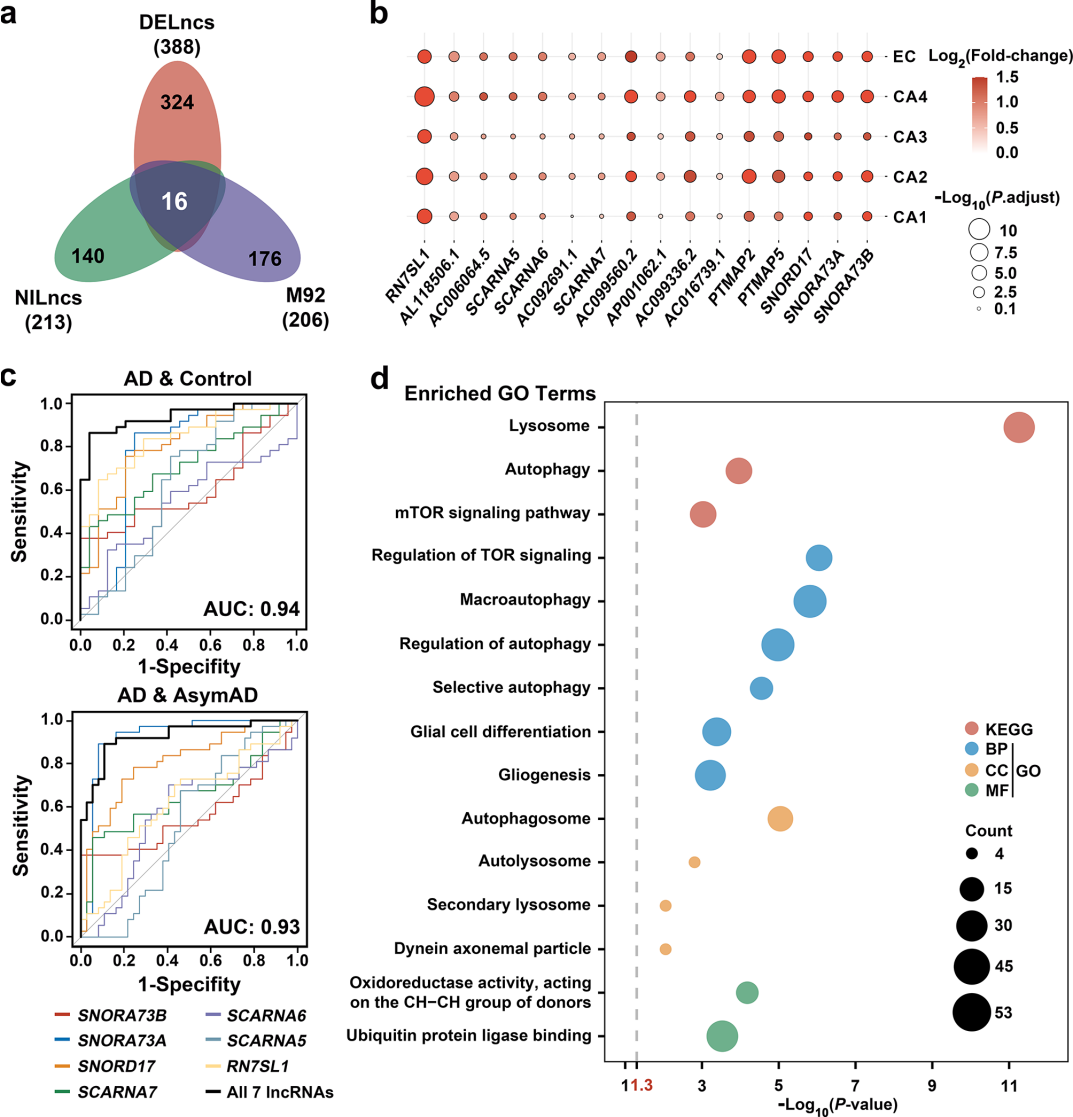
Identification and validation of key lncRNAs in AD pathology. (**a**) Venn diagram of lncRNAs within M92, DELncs, and NILncs, with 16 lncRNAs common to all three categories. (**b**) The extent of upregulation for the 16 common lncRNAs across all five subfields; the color represents the fold-change in pathological AD relative to controls, and the size of the circle represents the adjusted *P*-value (*P*.adjust). (**c**) ROC curves of the seven-lncRNA diagnostic model and seven single-lncRNA diagnostic models in distinguishing AD groups from healthy controls and AD from asymptomatic AD (AsymAD) groups of the GSE118553 dataset. AUC values for the seven-lncRNA diagnostic model in both test sets are shown. (**d**) GO and Kyoto Encyclopedia of Genes and Genomes (KEGG) pathway enrichment analysis of co-expressed genes of these seven lncRNAs. BP, biological process; CC, cellular component; MF, molecular function

As M92 was highly associated with neuroimmune processes and AD, an intersection analysis of the lncRNAs within M92, DELncs, and NILncs was performed. This led to the identification of 16 lncRNAs present across all three sets (Fig. 3a), exhibiting varying upregulation tendencies across the five subfields (Fig. 3b).

To validate the correlation between these 16 lncRNAs and AD, the expression matrix of an independent dataset (GSE118553) was obtained from the Gene Expression Omnibus [54]. In this dataset, “Asymptomatic AD (AsymAD) cases” refer to individuals who exhibited no clinical signs of dementia at the time of death but whose postmortem neuropathological evaluations revealed the characteristic pathologies of AD. “AD cases” pertain to individuals who were clinically and pathologically diagnosed with AD at the time of death. Conversely. “Control cases” describe individuals without any clinical dementia symptoms and lacking neuropathological indications of neurodegeneration. LncRNA expression data from the EC tissues of 37 AD patients, 37 AsymAD individuals and 24 control individuals in the GSE118553 dataset were utilized as the test set. Among the 16 lncRNAs, seven were detected in this test set. Using our lncRNA expression matrix, an SVM model was trained with these seven lncRNAs to classify patients with AD and healthy controls. This trained SVM model was then applied to classify samples in the test set. Encouragingly, the trained SVM model exhibited excellent performance, achieving an AUC of 0.94 for distinguishing patients with AD from healthy controls and an AUC of 0.93 for distinguishing AD from AsymAD samples (Fig. 3c).

GO and KEGG pathway enrichment analyses were performed on the linearly coexpressed mRNAs of these seven lncRNAs, which revealed a significant association of the seven lncRNAs with functions related to glial cell activity and cellular autophagy (Fig. 3d, Supplementary Material 2: Table S9).

Collectively, these findings highlight the relevance of the 16 identified lncRNAs, particularly the seven validated lncRNAs, in the context of AD pathology. The exceptional performance of the SVM model in an independent dataset further underscores the potential of these lncRNAs as biomarkers for distinguishing patients with AD from healthy controls. Furthermore, functional analyses suggested their involvement in critical processes associated with AD pathogenesis, such as glial cell function and cellular autophagy.

### *RN7SL1* upregulation in AD and its positive correlation with “ABC” scores

Next, we assessed the individual performance of the seven lncRNAs in distinguishing AD from controls and AD from AsymAD in the test set. According to the ROC curve analysis, *RN7SL1* exhibited the highest AUC value (AUC = 0.84) in distinguishing AD patients from healthy controls (Fig. 3c).

**Fig. 4:**
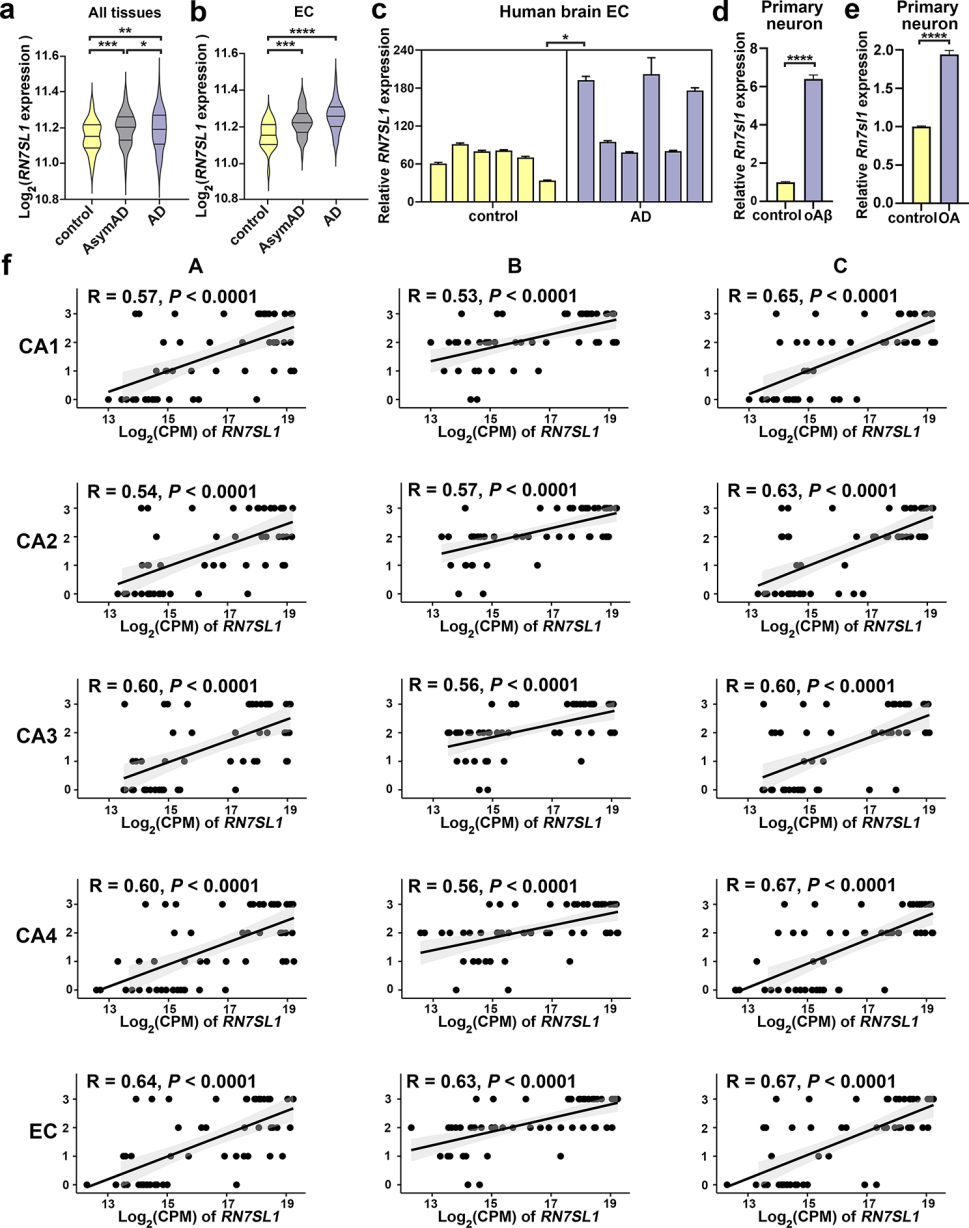
*RN7SL1* expression is upregulated in AD and positively correlates with “ABC” scores. (**a**, **b**) Using the GSE118553 dataset to validate *RN7SL1* expression, which progressively increases across control, AsymAD, and AD groups in all tissues (**a**) and the EC (**b**). (**c**) *RN7SL1* expression levels in the EC of brain tissues from individuals without AD pathology (control) and those with AD pathology were quantified using quantitative real-time reverse transcription PCR (qRT‒PCR) and normalized to *GAPDH* expression. Each group consisted of 6 donors, with 3 replicates per donor. (**d**) Expression of *Rn7sl1* 10 h after 10 µM oligomeric amyloid-β (oAβ) stimulation in rat primary neurons as determined by qRT‒PCR and normalized to *ACTB* expression (*n* = 3/group). (**e**) Expression of *Rn7sl1* 4 h after 10 nM okadaic acid (OA) stimulation in rat primary neurons as determined by qRT‒PCR and normalized to *ACTB* expression (*n* = 3/group). (**f**) Spearman correlation analysis of *RN7SL1* expression and “ABC” scores across five subfields. **A** Aβ plaque score; **B** Braak Neurofibrillary Tangle stage; **C** CERAD neuritic plaque score. Values represent mean ± standard error of mean (SEM). *P < 0.05, **P < 0.01, ***P < 0.001, and ****P < 0.0001, as determined by one-way analysis of variance (ANOVA; for comparing multiple groups) or Student’s *t*-test (for comparing two groups)

Moreover, within GSE118553, when specifically examining EC or considering all tissues combined, an increasing trend in *RN7SL1* expression was observed with the progression of AD pathology, implying that *RN7SL1* expression levels increase with the severity of AD pathology (Fig. 4a, b).

To experimentally validate differences in *RN7SL1* expression levels, qRT‒PCR was performed to evaluate *RN7SL1* expression in the EC tissues of six individuals with AD pathology and six individuals without AD pathology. *RN7SL1* expression was significantly greater in pathological AD than in controls (Fig. 4c). Further validation was then performed on the cellular model. OAβ, known for its neurotoxicity, is utilized as an in vitro cellular model for AD research and is applied to primary neurons [55]. After oAβ exposure, primary neurons cultured for 14 days in vitro exhibited a considerable increase in *Rn7sl1* expression (Fig. 4d). In addition, OA, an inhibitor of protein phosphatase-2 A, was used to stimulate neurons. OA treatment reportedly leads to tau hyperphosphorylation and neuronal death, representing a pathological model for AD [56]. Herein we observed an increase in *Rn7sl1* expression in primary neurons on stimulation with OA (Fig. 4e).

To explore the correlation between *Rn7sl1* expression and AD pathology, we examined the relationship between *Rn7sl1* levels and the severity of AD pathology, with the “ABC” score serving as a critical metric [57]. The “ABC” score includes the Aβ plaque score (A), Braak neurofibrillary tangle stage (B), and CERAD neuritic plaque score (C). There was revealed that an obvious positive correlation (with correlation coefficients R ranging from 0.53 to 0.67) between any of the “ABC” scores and *RN7SL1* expression (Fig. 4f), suggesting its role in AD pathology.

Overall, these findings provide functional evidence supporting the importance of the identified lncRNAs, particularly highlighting the role of *RN7SL1* in discriminating AD patients from healthy individuals. Moreover, the observed correlation between *RN7SL1* expression and AD pathology progression suggested its relevance to disease severity.

### *RN7SL1* deficiency impacts oAβ-induced microglial functions

Considering that *RN7SL1* was identified as an NILnc (Fig. 3a, b) and that its linearly coexpressed mRNAs are associated with glial cell activity (Fig. 3d), we investigated whether *RN7SL1* influenced neuroinflammation. Microglia, key contributors to neuroinflammation [36], were the focus of our study. Rat primary microglia and BV2 cells were used in these experiments. The purity of the primary microglia, confirmed by immunofluorescence staining, was 97% (Supplementary Material 1: Fig. S4a). In AD, microglia-mediated neuroinflammation is stimulated by Aβ. Therefore, we initially investigated the changes in the expression of *RN7SL1* in microglia following stimulation with oAβ. Primary microglia and BV2 microglial cell lines were treated with various concentrations of oAβ (0, 0.5, 1, 2, or 5 µM) for 3 h, and the qRT‒PCR results demonstrated that *Rn7sl1* expression initially increased and then decreased as the concentration of oAβ increased, with the peak expression of *Rn7sl1* observed at 1 µM oAβ (Supplementary Material 1: Fig. S4b, c). Similarly, when primary microglia and BV2 cells were stimulated with 1 µM oAβ for different durations (0, 1, 3, 6, or 12 h), the expression of *Rn7sl1* also showed an initial increase followed by a decrease, with the peak expression of *Rn7sl1* observed after treatment with oAβ for 3 h (Supplementary Material 1: Fig. S4b, c). This finding suggested that *Rn7sl1* expression is regulated by oAβ and exhibits dynamic changes in microglia.

**Fig. 5:**
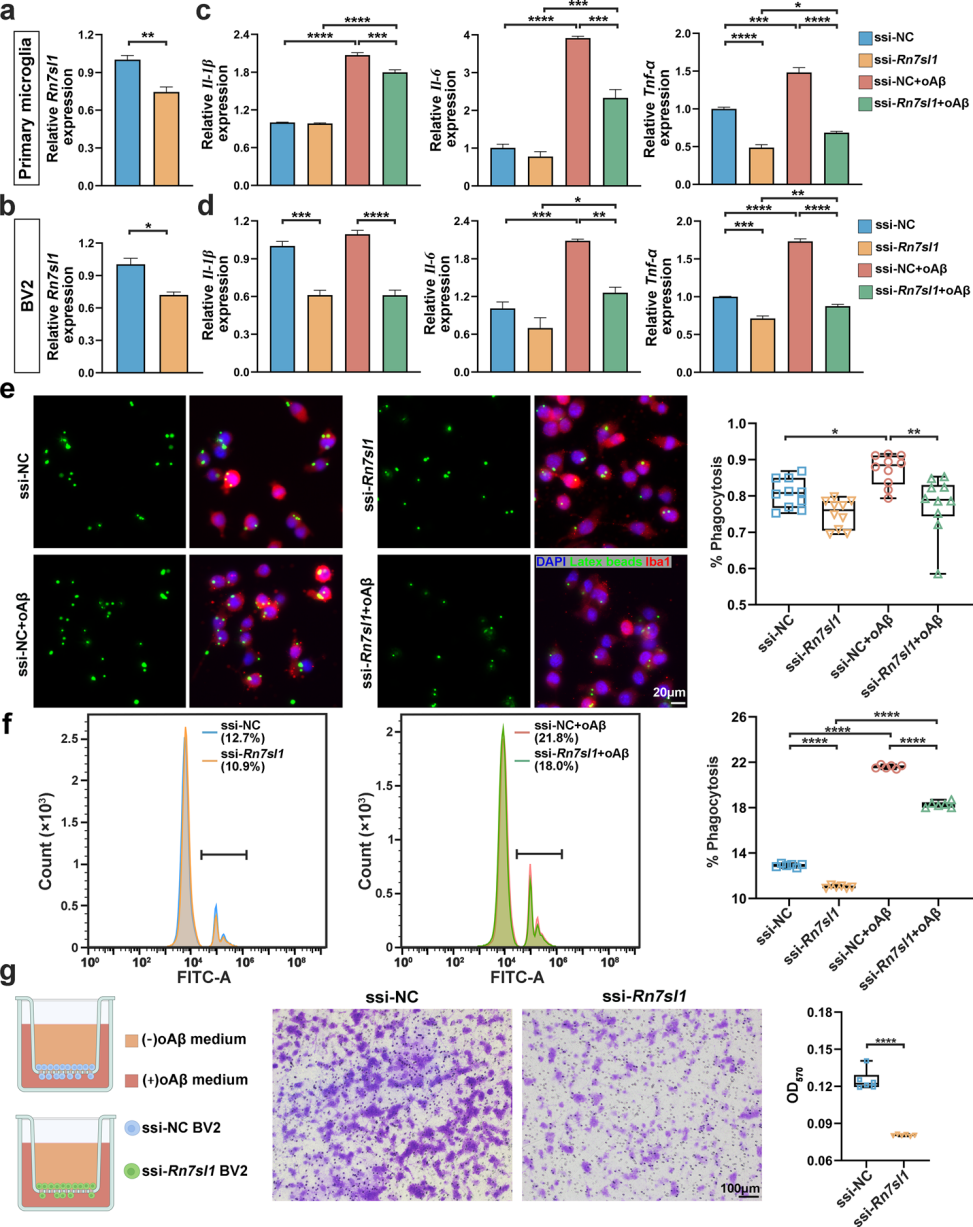
The impact of *RN7SL1* knockdown on neuronal apoptosis and morphology through microglia. (**a**, **b**) Knockdown efficiency of smart silencer (ssi) directed against *Rn7sl1* in rat primary microglia (**a**) and BV2 microglial cell lines (**b**), as determined by qRT‒PCR and normalized to *ACTB* expression. (**c**, **d**) Expression of proinflammatory cytokines in primary microglia (**c**) and BV2 cells (**d**) as determined by qRT‒PCR and normalized to *ACTB* expression. ssi-NC, cells treated with NC smart silencer as knockdown control; ssi-*Rn7sl1*, cells subjected to *Rn7sl1* knockdown using *Rn7sl1* smart silencer; ssi-NC + oAβ, knockdown control cells subjected to 5 µM oAβ stimulation for 24 h; ssi-NC + oAβ, knockdown control cells treated with 5 µM oAβ stimulation for 24 h; ssi-*Rn7sl1* + oAβ, *Rn7sl1*-knockdown cells treated with 5 µM oAβ stimulation for 24 h. (**e**) Representative immunofluorescence images of BV2 cells phagocytosing latex beads in four groups and phagocytosis rate statistics. % Phagocytosis represents the number of BV2 cells that phagocytosed latex beads divided by total number of IBA1^+^ (microglial marker) cells (*n* = 10/group). (**f**) Flow cytometry histogram showing the phagocytosis rate of BV2 cells in four groups. % Phagocytosis represents the percentage of FITC^+^ cells (cells that phagocytosed fluorescent beads) within the gated total cell population (*n* = 6/group). (**g**) Representative images showing the effects of *Rn7sl1* knockdown on BV2 cell migration. To quantify BV2 cell migration, absorbance at 570 nm was measured after crystal violet staining and washing (*n* = 6/group). Values represent mean ± SEM. *P < 0.05, **P < 0.01, ***P < 0.001, and ****P < 0.0001, as determined by one-way ANOVA (for comparing multiple groups) or Student’s *t*-test (for comparing two groups)

Subsequently, *Rn7sl1* was knocked down in primary microglia and BV2 cells using the lncRNA ssi (Fig. 5a, b). Persistent microglial activation and inflammatory cytokine secretion are recognized as drivers of neurodegeneration in AD pathogenesis [58]. Therefore, we stimulated primary microglia and BV2 cells with oAβ and assessed the impact of *Rn7sl1* knockdown. qRT‒PCR demonstrated that oAβ significantly upregulated the expression of the proinflammatory cytokines *Il *(interleukin) *-1β*, *Il-6*, and tumor necrosis factor (Tnf) -α in primary microglia, whereas *Rn7sl1* knockdown markedly reduced the expression of oAβ-induced proinflammatory cytokines (Fig. 5c). Similarly, oAβ stimulation significantly increased *Il-6* and *Tnf-α* expression in BV2 cells, and *Il-1β* expression tended to increase. However, *Rn7sl1* knockdown inhibited the oAβ-induced increase in proinflammatory cytokine expression, bringing the expression levels of these cytokines close to basal levels (Fig. 5d). The reduction in proinflammatory cytokine expression levels caused by *Rn7sl1* knockdown alone was not as significant as that caused by oAβ stimulation (Fig. 5c, d). These results suggested that *Rn7sl1* attenuates the activation of primary microglia and BV2 cells by reducing the oAβ-induced expression of proinflammatory cytokines.

Under pathological conditions, excessive phagocytosis by activated microglia is often observed. In microglial phagocytosis experiments, the phagocytic ratio represented the proportion of BV2 cells that engulfed latex beads out of the total cells (IBA1^+^ cells). The statistical results indicated that oAβ significantly promoted phagocytosis in BV2 cells, while *Rn7sl1* knockdown alleviated this phagocytic effect (Fig. 5e). This finding was further confirmed by flow cytometry data (Fig. 5f), where statistical analysis showed that *Rn7sl1* knockdown inhibited the increase in oAβ-induced phagocytic ability of BV2 cells.

To investigate whether *RN7SL1* affects microglial migration towards Aβ, Transwell assays were performed. Crystal violet staining indicated that *Rn7sl1* knockdown significantly inhibited microglial migration towards Aβ (Fig. 5g).

Collectively, these results revealed that *RN7SL1* deficiency mitigates the adverse effects of oAβ on microglia, including a reduction in oAβ-induced proinflammatory cytokines expression and phagocytosis, and attenuation of microglial migration towards Aβ.

### *RN7SL1* deficiency modulates neuronal morphology and functions through microglia

**Fig. 6:**
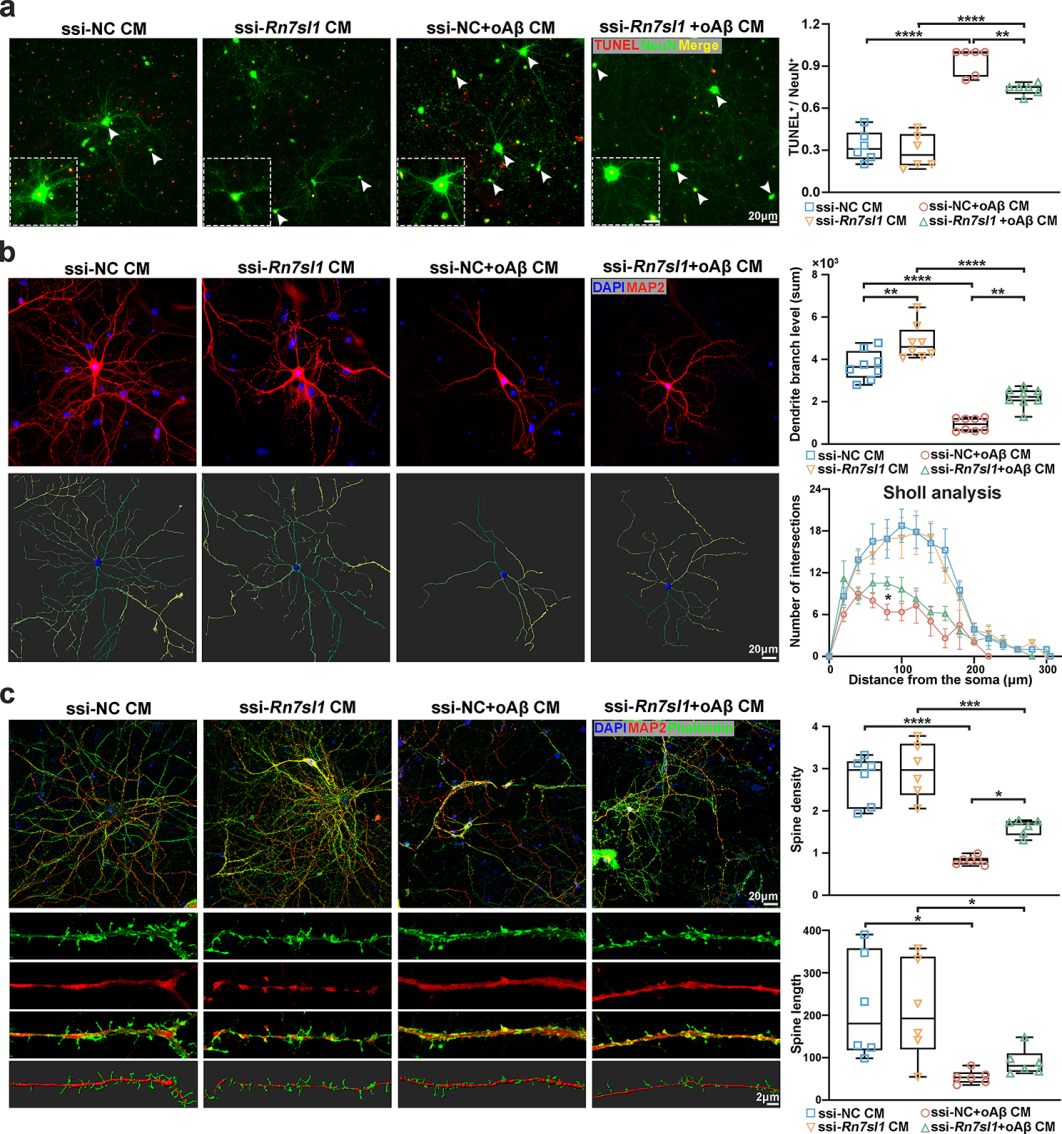
The impact of *RN7SL1* knockdown on neuronal apoptosis and morphology through microglia. (**a**) After stimulating primary neurons with primary microglial conditioned medium (CM) for 24 h, representative images depict apoptotic neurons identified by TUNEL assay (red) alongside NeuN staining (green; neuronal marker). Statistical results represent the ratio of TUNEL^+^ cells to NeuN^+^ cells (*n* = 6/group). (**b**) After stimulating primary neurons with primary microglial CM for 24 h, representative images were captured of the neurons immunostained with MAP2 (red) and 4′,6-diamidino-2-phenylindole (DAPI; blue). Three-dimensional reconstruction of the neurons was conducted using Imaris. Statistical analyses included dendrite branch level (*n* = 8/group) and neuron quantification through Sholl analysis (*n* = 8/group). (**c**) After stimulating primary neurons with primary microglial CM for 24 h, representative confocal microscopy images show the dendritic spines of the neurons immunostained with phalloidin (green) for spine visualization and MAP2 (red) for neuronal structure. Statistical analysis was conducted on dendritic spine density and length (*n* = 6/group). Values represent mean ± SEM. *P < 0.05, **P < 0.01, ***P < 0.001, and ****P < 0.0001, as determined by one-way ANOVA.

The activation of microglia and subsequent release of inflammatory cytokines in the brain can induce neuronal apoptosis and cytotoxicity. To further validate the impact of *RN7SL1* deficiency, we stimulated primary neurons with CM from different groups of primary microglia. TUNEL staining was used to detect neuronal apoptosis, and NeuN antibodies were used for neuron labelling. CM from oAβ-treated microglia was found to induce primary neuronal apoptosis; however, *Rn7sl1* knockdown mitigated this effect (Fig. 6a). Subsequently, morphological changes in the neurons were assessed. The dendrite branch levels were significantly reduced after oAβ CM treatment; ssi- *Rn7sl1* CM treatment alleviated this reduction (Fig. 6b). Furthermore, compared with those in the control group, the number of process intersections in the groups treated with ssi-*Rn7sl1* and oAβ CM increased according to Sholl analysis (Fig. 6b), suggesting an increase in neuronal complexity. We then performed phalloidin staining to explore the function of RN7SL1 in neuronal dendritic spines. The activation of microglia by oAβ significantly reduced the density and length of neuronal dendritic spines. However, when *RN7SL1* was knocked down in Aβ-induced microglia, after which the neurons were treated with CM, the dendritic spine density significantly increased compared to that in the Aβ control group, and the dendritic spine length tended to increase (Fig. 6c).

To explore whether *RN7SL1* deficiency could directly affect neurons, we conducted experiments involving oAβ stimulation and *Rn7sl1* knockdown in primary neurons. The qRT‒PCR result showed significant knockdown of *Rn7sl1* in primary neurons (Supplementary Material 1: Fig. S5a). Similar to experiments following microglial CM stimulation, TUNEL staining and immunofluorescence were used to assess neuronal apoptosis, morphology, and changes in dendritic spines. Stimulation with oAβ led to an increase in neuronal apoptosis (Supplementary Material 1: Fig. S5b), a decrease in dendrite branch level and complexity (Supplementary Material 1: Fig. S5c), and a reduction in dendritic spine density and total length (Supplementary Material 1: Fig. S5d). However, concurrent knockdown of *Rn7sl1* in the context of oAβ stimulation did not result in significant changes in these phenotypes (Supplementary Material 1: Fig. S5b, d). This indicates that *RN7SL1* does not directly act on neurons to cause their phenotypic changes.

Overall, these results indicated that *RN7SL1* depletion alleviates neuronal apoptosis and neurotoxicity caused by oAβ-induced microglia. This effect may be generated by influencing the activation of microglia and associated neuroinflammation.

## Discussion

Our results revealed significant differences in lncRNA expression profiles between individuals with AD pathology and without AD pathology across the five subfields. The majority of identified DELncs were associated with critical processes implicated in AD pathogenesis, such as synaptic function, cellular autophagy, neuroimmune responses, and glial cell function.

Synaptic degeneration is a fundamental aspect of AD pathology, and involves mechanisms triggered by the accumulation of toxic proteins, such as oAβ and tau, as well as potential synaptic elimination by glial cells [59]. Although some research has focused on lncRNAs related to synapses in AD, such as *BCYRN1*, which has been studied for its role in maintaining long-term synaptic plasticity by regulating local protein synthesis [60], further exploration of lncRNA involvement in this context is warranted.

The role of glial cells in AD is widely recognized. Notably, microglial proliferation and activation within the brain, particularly around Aβ plaques, are prominent characteristics of AD [24]. Astrocytes contribute directly to neuroinflammatory and neurodegenerative processes in AD [61], and lncRNAs mediate neuroinflammation in glial cells [62]. For example, *MEG3* overexpression has been reported to inhibit astrocyte activation, reduce inflammatory injury, and alleviate neuronal damage [63].

In this study, GO analysis indicated the enrichment of neuroimmune processes, which play a large role in AD pathogenesis [64]. Several studies have explored the involvement of lncRNAs in neuroimmune processes in AD. For instance, Tan et al. identified 17 lncRNAs, including *LINC00507*, involved in competing endogenous RNA networks influencing ferroptosis-related molecular patterns and immune characteristics in AD [65]. Li et al. also identified lncRNAs, including *LINC00472*, that participate in immune pathways related to CD8 + T cells, thereby influencing AD pathology [66].

Considering the crucial role of neuroimmune processes in AD and based on our differential expression analysis, we identified a set of NILncs encompassing all 13 shared DELncs across the five subfields. Among them, 16 lncRNAs were present in the M92 module and the DELnc and NILnc sets. At present, research on these 16 lncRNAs is relatively scarce. However, owing to their strong ability to distinguish AD patients from healthy controls in other datasets, these lncRNAs, particularly the seven lncRNAs validated in the GSE118553 dataset, were investigated. Subsequent studies exploring the expression levels of these lncRNAs in blood may further reveal their potential in early diagnosis and pathological scoring.

Among the seven validated lncRNAs, *RN7SL1*, a structured non-coding RNA, exhibited the greatest ability to discriminate patients with AD from healthy controls. We then investigated its potential role in neuroinflammatory responses in AD. *RN7SL1* has demonstrated remarkable evolutionary conservation and serves as a constituent of the signal recognition particles, linking nascent protein synthesis to proper membrane localization [67]. CAR-T cells expressing *RN7SL1* have been found to enhance autonomous and endogenous immune function, improving solid tumour treatment outcomes [68]. However, the role of *RN7SL1* in neuroinflammation and AD remains to be investigated. We found that *RN7SL1* is a DELnc in AD and an NILnc and that it plays a key role in regulating microglial functions.

Microglia play a dual role in AD development, serving as both protectors against and contributors to disease progression. These cells phagocytose cellular debris and clear Aβ aggregates, maintaining brain tissue homeostasis to potentially prevent AD onset [58]. However, the continuous accumulation of toxic amyloid species induces chronic inflammation in microglia [69]. In this inflammatory state, microglia exhibit heightened phagocytosis and release neurotoxic cytokines, which may paradoxically contribute to AD [21]. In this study, we explored the impact of Aβ stimulation on microglia, revealing increased expression of proinflammatory cytokines, increased phagocytic activity, and consequential neuronal apoptosis and morphological changes. Notably, *RN7SL1* deficiency alleviated microglial activation induced by Aβ, reduced proinflammatory cytokine expression, and enhanced phagocytosis, thereby mitigating neuronal damage. These findings indicate that *RN7SL1* predominantly exerts adverse effects on microglia, driving them into an activated and inflammatory state with the potential for neurotoxicity. Consequently, *RN7SL1* may emerge as a promising regulatory factor in AD.

Surprisingly, we found that *RN7SL1* enhanced the migration of microglia towards Aβ. In conjunction with the observed phagocytic effects, under normal physiological conditions, *RN7SL1* is hypothesized to promote the clustering of microglia and the phagocytosis of Aβ plaques. However, as toxic amyloid species continuously accumulate, *RN7SL1* induces the transition of microglia to an inflammatory state, ultimately leading to neuronal damage.

In addition to the influence of *RN7SL1* on microglial function, its cis-target genes have also been reported to be associated with microglia and inflammation. Li et al. discovered that m^6^A modification of *POLE2* within the microglial m^6^A epitranscriptome is associated with an anti-inflammatory microglial phenotype [70]. Through single-cell RNA sequencing, *RPS29* has been reported to be associated with the function of microglia during development [71, 72]. Furthermore, *MGAT2* and *ARF6* are involved in the regulation of inflammation [73, 74]. However, the interaction between *RN7SL1* and its target genes, as well as its impact on neuroinflammation and microglial function, requires further mechanistic exploration.

This study has certain limitations. First, in this study, the PMI of brain tissue samples from two donors was more than 48 h, and some samples had low RIN values, which could impact the lncRNA transcriptome. To minimize such potential confounding effects, the PMI and RIN, along with age and area, were included as covariates at the initiation of surrogate variable analysis and linear modelling, before proceeding with subsequent bioinformatics analyses. Second, although we investigated the influence of *RN7SL1* on microglial function, the precise underlying mechanisms remain elusive. Third, to simulate an AD cell model, we treated neurons and microglia with Aβ; however, this in vitro model only mimicked the state of acute injury and may not fully reflect cellular changes under the actual conditions of AD. Further studies are thus warranted to address these shortcomings.

## Conclusions

Our results highlight the fundamental role of lncRNAs in the intricate landscape of AD pathology. Through comprehensive bioinformatics analysis, we identified key lncRNAs, notably *RN7SL1*, revealing their substantial involvement in crucial processes central to AD. The valid role of *RN7SL1* in modulating neuroimmune responses, particularly its influence on microglial functions, highlights its potential as a therapeutic target in AD intervention. Further investigations are imperative to elucidate the precise mechanisms through which these lncRNAs exert their effects on AD.

### Electronic supplementary material

Below is the link to the electronic supplementary material.

Supplementary Material 1

Supplementary Material 2

## Data Availability

Raw RNA-Seq data are available from the corresponding authors on reasonable request.

## Abbreviations

AD: Alzheimer’s Disease
ANOVA: analysis of variance
AsymAD: asymptomatic AD
AUC: area under the curve
CA: cornu ammonis
CM: conditioned medium
CNS: central nervous system
CPM: counts per million
DAM: disease-associated microglia
DAPI: 4’,6-diamidino-2-phenylindole
DELncs: differentially expressed lncRNAs
DMEM: Dulbecco’s modified eagle medium
EC: entorhinal cortex
GO: Gene Ontology
Il: interleukin
KEGG: Kyoto Encyclopedia of Genes and Genomes
lncRNA: long non-coding RNA
NILnc: neuroimmune-related lncRNA
OA: okadaic acid
oAβ: oligomeric amyloid-β
PCA: principal component analysis
PMI: postmortem interval
qRT‒PCR: quantitative real-time reverse transcription PCR
RIN: RNA integrity number
ROC: receiver operating characteristic
ssi: smart silencer
SVM: support vector machine
Tnf: tumor necrosis factor
WGCNA: weighted gene co-expression network analysis
