# Supplementary material for: Profiling of long non-coding RNAs in hippocampal–entorhinal system subfields: impact of RN7SL1 on neuroimmune response modulation in Alzheimer’s disease
Source: J Neuroinflammation. 2024 Apr 6;21:84. doi: 10.1186/s12974-024-03083-x (PMC10999094; doi:10.1186/s12974-024-03083-x)
Supplement: Supplementary file 1 — Supplementary Material 1 [file 12974_2024_3083_MOESM1_ESM.docx]

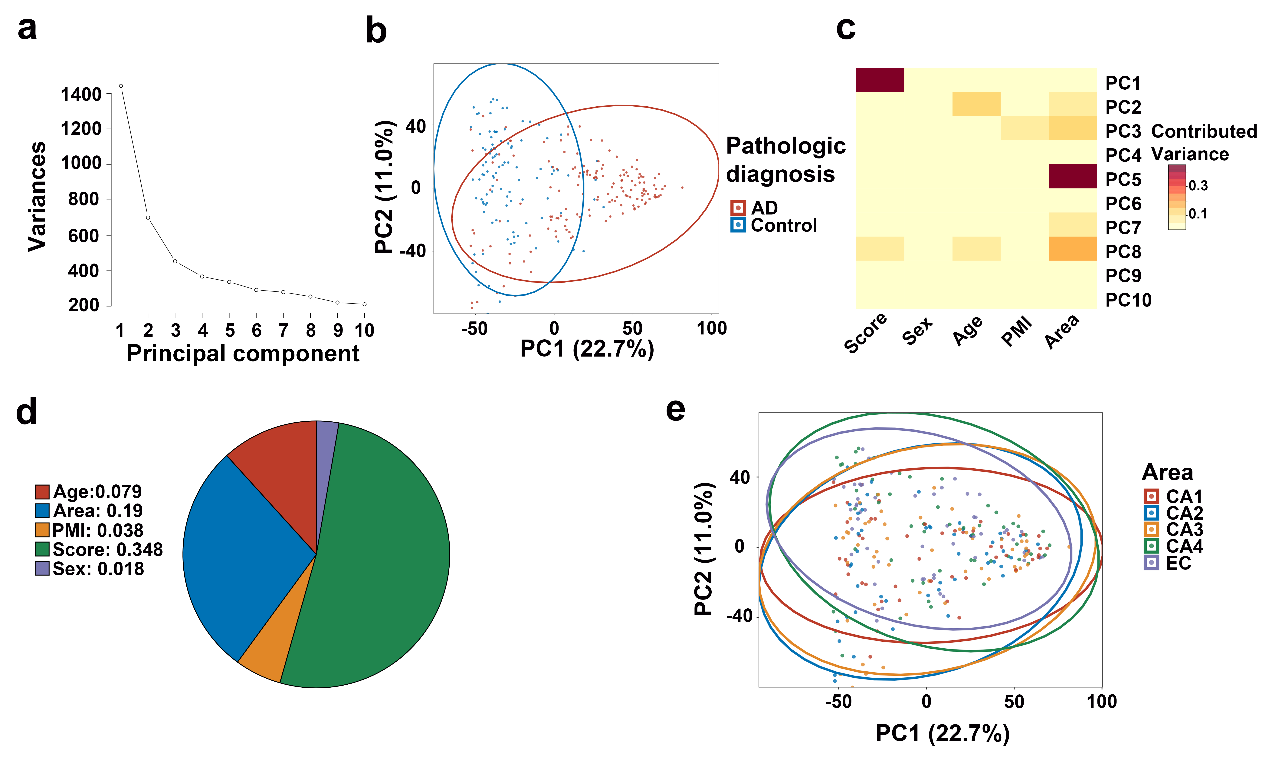


**Fig. S1 Principal component analysis (PCA) results of lncRNA expression pattern.** (a) Variance of top 10 principal components. (b, e) PCA plot of hippocampal–entorhinal system subfield RNA-seq data: (b) AD (N = 155), control (N = 107); (e) CA1 (N = 50), CA2 (N = 53), CA3 (N = 53), CA4 (N = 53), and EC (N = 53). (c) Heatmap of contributed variance of five covariates to the top 10 principal components. Color bar represents contributed variance. (d) Pie chart showing the ratio of each trait’s contribution to transcriptomic variance. CA1–4, cornu ammonis subfields 1–4; EC, entorhinal cortex.


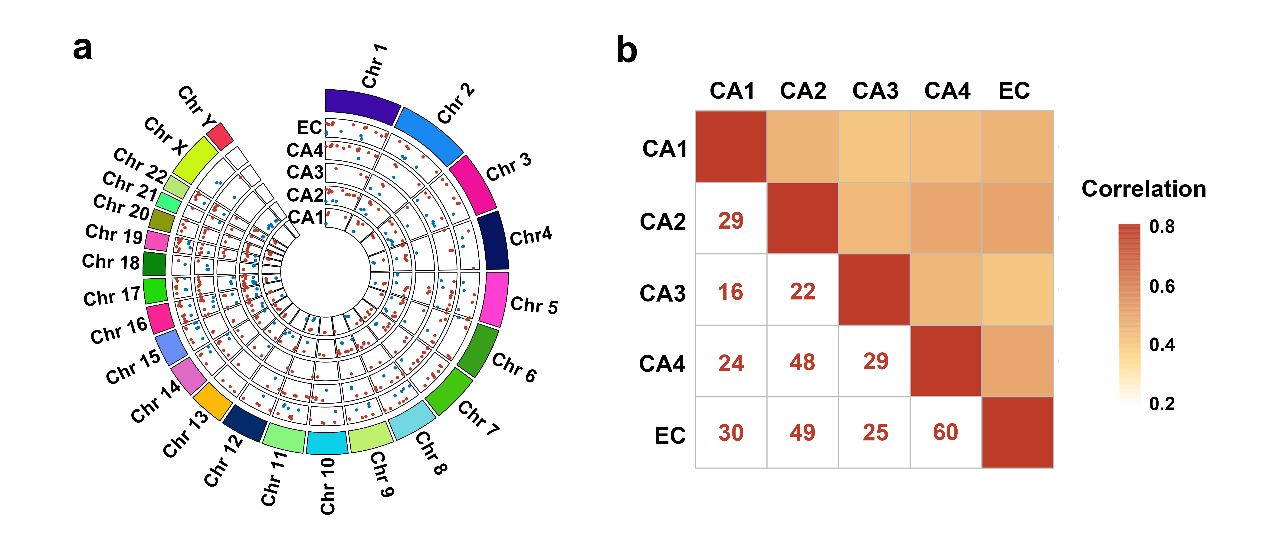


**Fig. S2 Patterns of differentially expressed lncRNAs (DELncs).** (a) Chromosome position of DELncs in each subfield. Blue dots indicate downregulated lncRNAs, and red dots indicate upregulated lncRNAs. (b) Pairwise correlation of DELncs from the five subfields. Numbers represent the number of shared DELncs. Color bar shows Pearson correlation between fold changes of all lncRNAs from two subfields.


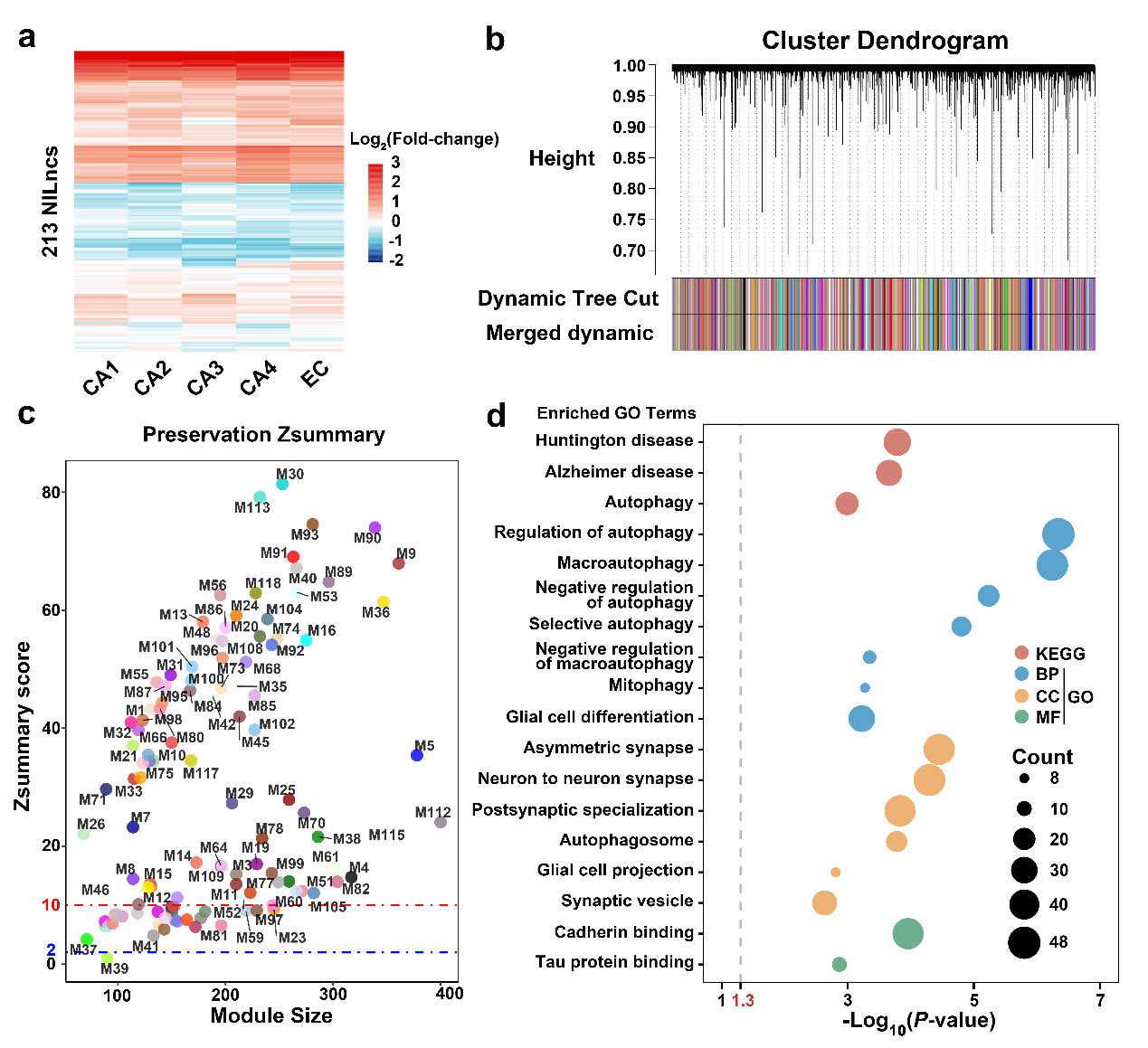


**Fig. S3 Identification of neuroimmune-related lncRNAs (NILncs) and WGCNA.** (a) Differential expression patterns of NILncs across the five subfields, with colors representing log_2_(Fold-change) of each lncRNA. (b) Clustering dendrogram of all 22,418 lncRNAs with dissimilarity based on topological overlap with assigned module colors below. The gray module was categorized as the ‘bin’ module containing genes not assigned to any module. (c) Module preservation statistics. A Zsummary score of >10 indicated a strongly preserved module, <2 indicated a non-preserved module, while a score between 2 and 10 indicated a moderately preserved module. (d) Gene ontology (GO) and Kyoto Encyclopedia of Genes and Genomes (KEGG) pathway enrichment analysis of co-expressed genes of lncRNAs within M92. BP, biological process; CC, cellular component; MF, molecular function.

**
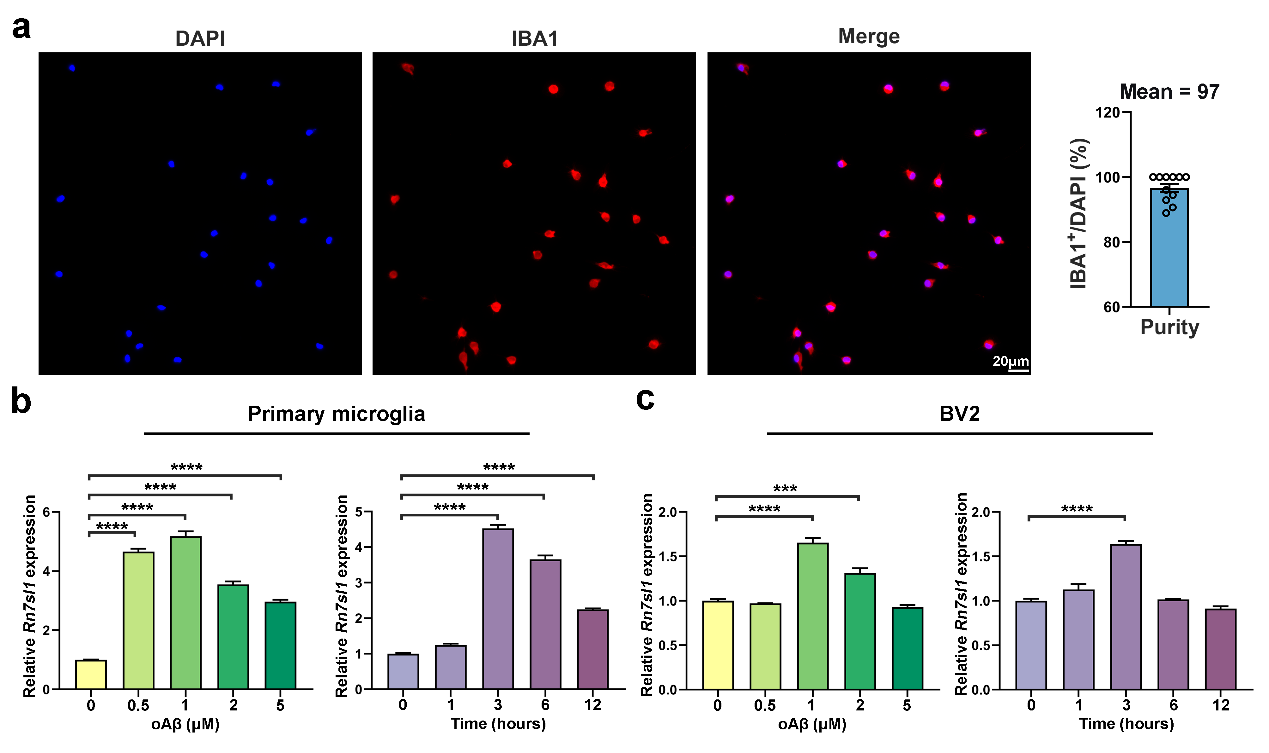
**

**Fig. S4** **Dynamic response of *RN7SL1* expression in microglia to oligomeric amyloid-β (oAβ) stimulation.** (a) Purity of rat cultured primary microglia. Representative images of rat primary microglia immunostained with the microglial marker IBA1 (red) and 4′,6-diamidino-2-phenylindole (DAPI; blue). Purity of microglia was defined as the ratio of IBA1^+^ cells to total cells and determined by averaging the results from 10 fields of view; mean purity was 97%. (b) Expression of *Rn7sl1* in primary microglia after stimulation with different concentrations of oAβ (0, 0.5, 1, 2, 5 μM) for 3 h and across different time points (0, 1, 3, 6, 12 h) with 1 μM oAβ, as measured by quantitative real-time reverse transcription PCR (qRT‒PCR) and normalized to *ACTB* expression (n = 3/group). (c) Expression of *Rn7sl1* in BV2 microglial cell lines after stimulation with different concentrations of oAβ (0, 0.5, 1, 2, 5 μM) for 3 h and across different time points (0, 1, 3, 6, 12 h) with 1 μM oAβ, as measured by qRT‒PCR (n = 3/group). Values represent mean ± standard error of mean (SEM). ****P* < 0.001, and *****P* < 0.0001, as determined by one-way analysis of variance (ANOVA).


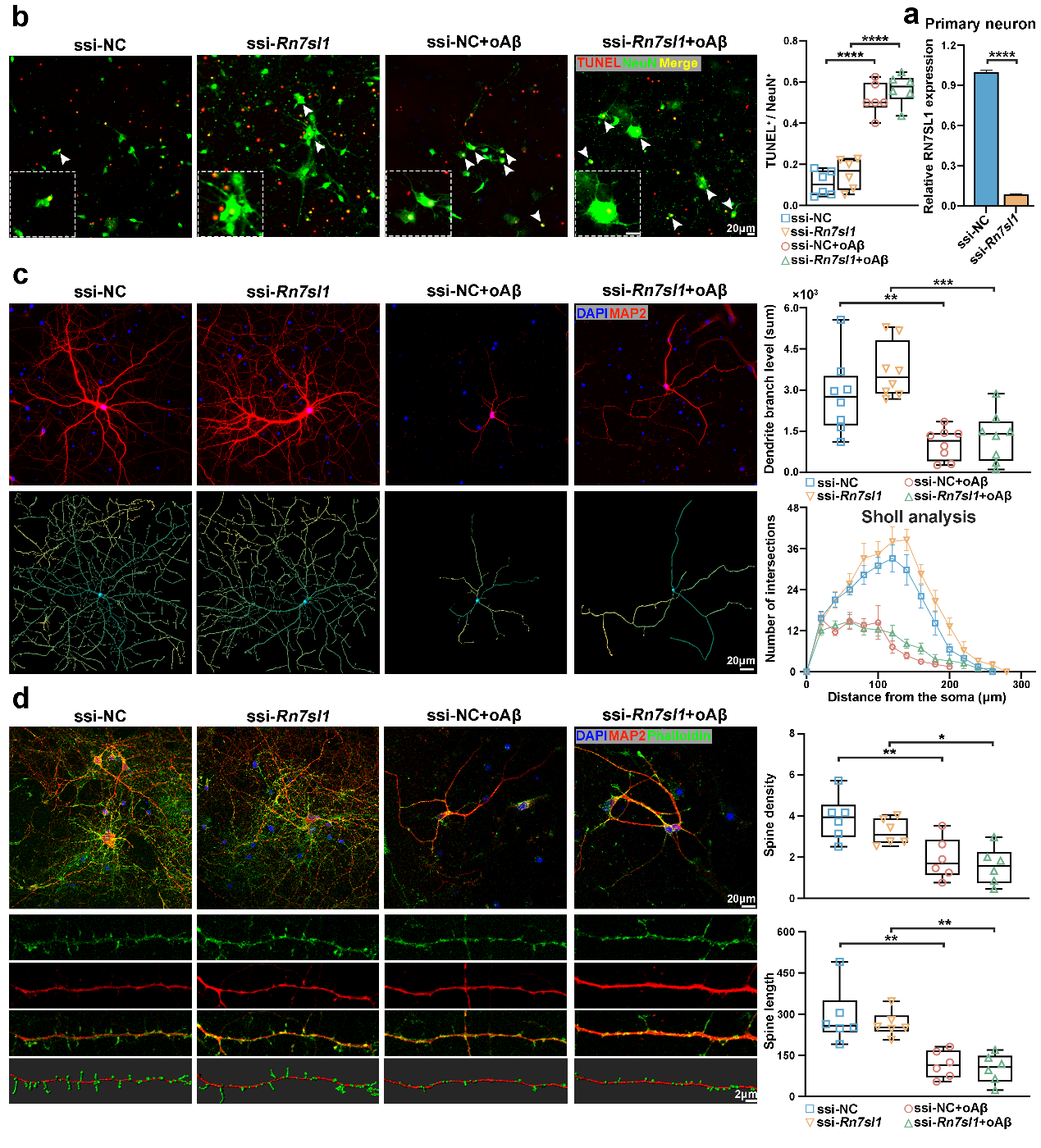


**Fig. S5 The impact of *RN7SL1* knockdown on apoptosis and morphology in neurons.** (a) Knockdown efficiency of smart silencer (ssi) directed against *Rn7sl1* in rat primary neurons as determined by qRT‒PCR and normalized to *ACTB* expression. (b) Representative images depict apoptotic primary neurons identified by TUNEL assay (red) alongside NeuN staining (green; neuronal marker) in four groups. Statistical results represent the ratio of TUNEL^+^ cells to NeuN^+^ cells (n = 6/group). ssi-NC, neurons treated with NC smart silencer as knockdown control; ssi-*Rn7sl1*, neurons subjected to *Rn7sl1* knockdown using *Rn7sl1* smart silencer; ssi-NC+oAβ, knockdown control neurons subjected to 10 μM oAβ stimulation for 6 h; ssi-NC+oAβ, knockdown control neurons treated with 10 μM oAβ stimulation for 6 h; ssi-*Rn7sl1*+oAβ, *Rn7sl1*-knockdown neurons treated with 10 μM oAβ stimulation for 6 h. (c) Representative images of primary neurons immunostained with MAP2 (red) and DAPI (blue) in four groups. Three-dimensional reconstruction of neurons was conducted using Imaris. Statistical analyses included dendrite branch level (n = 8/group) and neuron quantification through Sholl analysis (n = 8/group). (d) Representative confocal microscopy images show the dendritic spines of the neurons immunostained with phalloidin (green) for spine visualization and MAP2 (red) for neuronal structure. Statistical analysis was conducted on dendritic spine density and length (n = 6/group). Values represent mean ± SEM. **P* < 0.05, ***P* < 0.01, ****P* < 0.001, and *****P* < 0.0001, as determined by one-way ANOVA.
